# A High-Precision Protocol for Identification of Preschool Children at Risk for Persisting Obesity

**DOI:** 10.1371/journal.pone.0000535

**Published:** 2007-06-20

**Authors:** Toomas Timpka, Marianne Angbratt, Per Bolme, Göran Hermansson, Anders Häger, Lars Valter

**Affiliations:** 1 Section of Social Medicine and Public Health, Department of Health and Society, Linköping University, Linköping, Sweden; 2 Centre for Public Health, Östergötland County Council, Linköping, Sweden; 3 Department of Paediatrics, Linköping University Hospital, Linköping, Sweden; James Cook University, Australia

## Abstract

**Background:**

Recent studies suggest that adolescent adiposity is established already in preadolescence. Earlier studies have confirmed a strong tracking of obesity from adolescence to adulthood. Our aim was to examine the diagnostic accuracy of a population-derived protocol for identification of preschool children at risk for obesity in preadolescence.

**Methodology/Principal Findings:**

We analysed data obtained for child health surveillance up to age 5 from 5778 children born in a Swedish county in 1991. The basic data set included age, sex, and weight and height measurements from the regular checkups between ages 1.5 and 5. Data not routinely collected in the child health centre setting were disregarded. The children were at age 10 randomly assigned to protocol derivation and validation cohorts and assessed for obesity according to IOTF criteria. The accuracy of predicting obesity in the validation cohort was measured using decision precision, specificity, and sensitivity. The decision protocol selected 1.4% of preschool children as being at obesity risk. The precision of the protocol at age 10 was 82% for girls and 80% for boys, and the specificity was 100% for both boys and girls. The sensitivity was higher for girls (41%) than for boys (21%). The relative risk for obesity at age 10 estimated by the odds ratio for individuals selected by the protocol compared to non-selected peers was 212.6 (95% confidence interval 56.6 to 798.4) for girls and 120.3 (95% CI 24.5 to 589.9) for boys.

**Conclusion/Significance:**

A simple and inexpensive decision protocol based on BMI values proved to have high precision and specificity for identification of preschool children at risk for obesity persisting into adolescence, while the sensitivity was low especially for boys. Implementation and further evaluations of the protocol in child health centre settings are warranted.

## Introduction

The prevalence of childhood obesity and overweight is rapidly increasing worldwide,[1] and the North European countries are no exception.[2], [3] Recent studies have suggested that major development of adiposity in young adults is fully established. by age 11 [4], [5] This fact, that obesity evident in preadolescence indicates obesity in early adulthood with a high degree of accuracy, implies that preschool detection of obesity risk along with prevention at school age are becoming increasingly important. Nevertheless, despite that a high proportion of obese preschool children remain obese after puberty, other obese children spontaneously regain normal weight before adolescence [6], [7], [8]. There are few means available for identification of the high risk children in order to provide them with focused treatment programs.

Methods for standardizing the collection and interpretation of clinical data using data-generated indices and rating scales are today well established,[9] BMI, calculated as weight (kg)/height (m) squared, has been used to define morbidity-related cut-off points for obesity and overweight among adults.[10] Among children, the index has been established for defining obesity and overweight in individuals at specific ages,[11], [12] but these cut-off points have not been developed to predict the persistence of these conditions at an individual level. Practice studies have also shown that clinicians use a “total impression,” rather than BMI charts, to identify children at risk for persisting obesity.[13] The aim of this study is to examine the diagnostic accuracy of a clinical protocol derived from longitudinal total population data for identification of preschool children at risk for persisting obesity. The term “persisting obesity” refers to obesity persisting into young adulthood, based on the recently reported trackings of obesity from preadolescence and adolescence to adulthood. To make the protocol clinically applicable and easy to use, only data available from child health centres obtained from children prior to school start were used for the protocol development. The study is part of the “Barn är viktiga” (Children are important, Swedish only) programme aimed at preventing obesity in children and adolescents by evidence-based strategies at individual and population levels in Östergötland county (pop. 420,000), Sweden. The ambition is that the children selected by the protocol will be provided with family-based interventions by the school health services.[14], [15]

## Methods

The primary study population consisted of all children born in Östergötland in 1991 (n = 5 778; 3 030 boys and 2 748 girls). The analysis was based on the present Swedish system which entails that almost all children at child health centres are examined and screened for weight and height at regular intervals. On the average, each child pays 20 visits to the child health nurse, and three visits to a General Practitioner before age 6. Data were accordingly collected from the population between 1991 and 2001, excluding children in families moving out from the county. Parents were informed of the study by a letter and by advertisements in the local newspaper. Those families not wanting their children's data to be included in the study were asked to notify their child health centre or the research group to have their research record erased. Before initiation, the Ethics Committee at Linköping University approved the study design.

### Data collection

The weight and height of the children in the study population were measured at routine checkups at 1½, 2½ , 4, 5, and 10 years of age. All participating nurses received a training programme before each data collection period. Validated scales were used for all measurements. Weight was measured to the nearest 0.1 kg and length to the nearest 0.1 cm. The data were first recorded during scheduled visits at the child health centres (ages 1½ to 5) and thereafter at visits to the school health service (age 10). About 60% of the child health centres in the county measured the children at age 5±3 months and approximately 40% of the children at age 5½±3 months. The children were measured by school health services in the fourth grade, when the children were between ages 9.7 and 11.3 years. The International Obesity Task Force (IOTF) criteria were used to define obesity.[12] These criteria identify BMI values for each age associated with a predicted BMI of 30 at the age of 18. Age-specific BMI (weight in kg divided by height in m^2^) was calculated for each individual using the nearest tabulated age method. For the obesity prevalence analyses, a ±3 months age interval was defined for the checkups at 2½, 4, and 5 years of age. Children examined outside these intervals were excluded from the analyses. The final study population consisted of children born in 1991 who were still living in Östergötland in January, 2001 (n = 5105). The BMI distribution and prevalence of obesity at the different well-child checkup ages are shown in table 1.

**Table 1:** Mean BMI and prevalence of obesity according to IOTF definitions at child health centre checkups and at age 10.

	*Boys*							*Girls*						
	n	BMI cut	Age		BMI		Obesity prevalence	n	BMI cut	Age		BMI		Obesity prevalence
Checkup			mean	SD	mean	SD	percent			mean	SD	mean	SD	percent
2½ years	1811	19.8	2.54	0.08	16.73	1.39	2.5	1671	19.5	2.54	0.08	16.44	1.37	2.0
4 years	2044	19.3	4.05	0.07	16.09	1.34	1.9	1900	19.1	4.05	0.07	15.94	1.42	2.6
5 years	2084	19.3	5.25	0.25	15.92	1.47	2.4	1929	19.2	5.25	0.25	15.92	1.47	3.4
10 years	2322	24.0	10.50	0.35	18.33	3.12	4.8	2147	24.1	10.49	0.34	18.23	3.05	3.9

BMI cut: Age- and sex specific IOTF cut-off value for obesity.

### Statistical analyses

**Figure 1:**
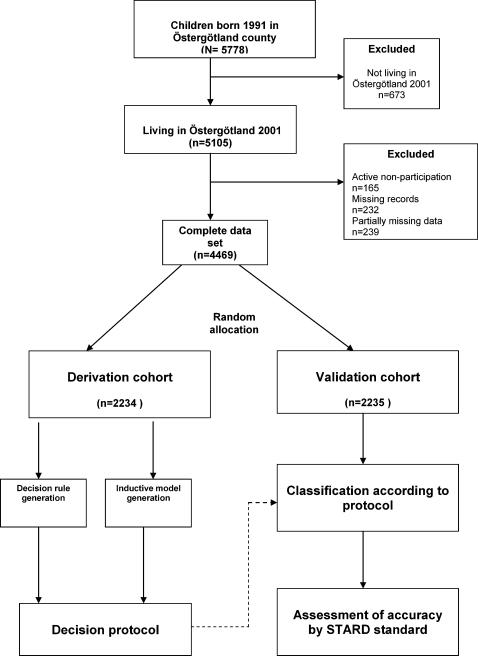
Study design overview.

The variables used for the analyses included sex, BMI at the ages 1½, 2½, 4, and 5 years. In addition, we took into account validated evidence related to childhood growth, BMI and development of persisting obesity by constructing variables from the concept of adiposity rebound .[16](age at adiposity rebound, BMI at adiposity rebound, and early adiposity rebound) and rapid childhood BMI increase.[17] Early adiposity rebound was defined by the age at which the BMI curve increased after a recess during the first year. The children were ranked by their age at the time of the increase, and the 20% of the children with the earliest rise (at age 4.0 years or earlier) were defined as having an early adiposity rebound. BMI increase was calculated as the relative difference between BMI at age 1.5 and 5 years. A random-number generation algorithm was used to divide the children who completed the data collection in 2001 into two equally large groups; the derivation cohort and the validation cohort (figure 1). The derivation cohort was used to generate a set of decision rules[18], [19] based on international criteria for obesity, and an inductive decision model[20] based on a set of variables constructed from weight and height measurements from the regular checkups between ages 1.5 and 5. The decision rules and the model were optimized to predict the likelihood of obesity at age 10 from weight and height patterns during pre-school. Due to that the introduction of the decision protocol was not allowed to cause significant extra costs, data not regularly collected in the child health centre setting were disregarded. In the validation step of the analysis, the validation cohort was used to assess the diagnostic accuracy of the protocol according to Standards for Reporting of Diagnostic Accuracy (STARD) criteria.[21], [22] All statistical analyses were made in SPSS v11.5. For those children with missing values not exceeding one measurement, the nearest neighbour imputation was used in the longitudinal analyses.

#### Construction of decision protocol

When determining the final decision protocol, the clinical epidemiological requirements on the combination of decision rules and BMI cut-off values at age 5 were that there should be a sensitivity of at least 20%, a specificity of 98%, and a precision of at least 80% for both boys and girls. Previous attempts to develop child adiposity intervention protocols have shown that low precision results in a protocol with limited clinical value.[23] Consequently, our approach was to prioritise high precision and specificity in order to avoid unnecessary interventions, and to aid increasing compliance among children and parents selected by the protocol.

#### Decision protocol validation

The diagnostic accuracy of the decision protocol was assessed according to the STARD procedure on validation.[21] The classification performance was assessed by calculating the precision of the decision to treat, sensitivity, specificity, and odds ratios. To further assess the accuracy of the decision rule component in the protocol, it was also compared to the two second-best rules. When examining the inductive model component, the cut-off point at BMI 20 was also compared to the cut-off points representing BMI 16 to 19.

## Results

### Decision rules based on definitions of obesity

Table 2 shows the odds ratios for univariate regression analyses using derivation cohort data of associations between obesity at 10 years of age and the corresponding variable. For the development of a decision rule based on categorical variables, the three best performing variables in the univariate analyses were chosen. These variables (obesity at the ages of 2.5 years, 4 years and 5 years, respectively) were analysed both separately and combined in pairs. The classification performance of this set of decision rules was assessed by calculating the precision, sensitivity, specificity, and odds ratio at 95% confidence intervals. The three best performing decision rules identified using the derivation cohort data were considered for inclusion in the decision protocol (table 3).

**Table 2:** Univariate logistic regression analyses using derivation cohort data with regard to association to obesity at age 10.

	Boys		Girls		Total	
Variable	OR	95% CI	OR	95% CI	OR	95% CI
Sex					1.37	0.92–2.05
BMI at 1½ years	1.81	1.49–2.20	1.67	1.35–2.06	1.75	1.51–2.02
BMI at 2½ years	2.63	2.08–3.33	2.27	1.69–3.05	2.52	2.11–3.02
BMI at 4 years	2.81	2.24–3.52	2.56	2.07–3.17	2.66	2.28–3.11
BMI at 5 years	2.99	2.40–3.73	2.96	2.33–3.76	2.90	2.48–3.39
Obesity at 2½ years	17.2	7.1–41.8	10.0	2.6–38.5	15.0	7.3–31.0
Obesity at 4 years	35.2	14.2–87.3	32.2	14.2–73.1	31.6	17.3–57.4
Obesity at 5 years	61.3	26.1–144.3	76.4	33.7–173.2	62.2	35.0–110.5
Early adiposity rebound	1.35	0.70–2.62	2.20	1.00–4.81	1.60	0.97–2.64
Age at adiposity rebound	0.67	0.54–0.84	0.53	0.38–0.74	0.63	0.53–0.75
BMI at adiposity rebound	3.43	2.62–4.48	2.86	2.12–3.86	3.17	2.60–4.86
BMI change (%)	1.19	1.15–1.24	1.17	1.13–1.22	1.17	1.14–1.20

OR = odds ratio

95% CI = 95% confidence interval

Early adiposity rebound = Adiposity rebound before age 5 (y/n)

Age at adiposity rebound = Adiposity rebound at age 2½ years, 4 years or at age 5 years and later.

BMI change = BMI at 5 years/BMI at 1½ years

**Table 3:** Diagnostic accuracy of the three best performing decision rules extracted from the derivation cohort for selection of children at risk for persisting obesity at the 5-year checkup. All other rule combinations displayed poorer decision preformance.

Decision rule	Fraction chosen for treatment (%)	Sensitivity (%)	Specificity (%)	Precision of treatment choice (%)	OR	95% CI
**All (n = 1860)**						
Obesity at 4- and 5-year checkups	1.9	29	99	69	66.82	31.43–142.08
Obesity at either 4 or 5-year checkup	4.0	46	98	53	42.15	24.51–72.49
Obesity at 5-year checkup	3.2	44	99	62	58.72	32.41–106.37
**Girls (n = 913)**						
Obesity at 4- and 5-year checkups	2.2	33	99	60	54.31	20.34–145.04
Obesity at either 4 or 5-year checkup	4.6	58	98	50	57.07	25.86–125.93
Obesity at 5-year checkup	3.7	53	98	56	64.23	28.01–147.28
**Boys (n = 947)**						
Obesity at 4- and 5-year checkups	1.7	27	100	81	107.73	29.34–394.84
Obesity at either 4 or 5-year checkup	3.4	37	98	56	36.66	16.73–80.37
Obesity at 5-year checkup	2.7	37	99	69	64.60	26.09–159.93

OR = odds ratio

95% CI = 95% confidence interval

### Inductive decision model

Using derivation cohort data, an inductive model was also developed for the prediction of obesity at the age of 10 years. The variables considered for inclusion in a stepwise logistic regression analysis were sex and those from the univariate analyses found to have been associated (p<0.05) with obesity at the age of 10 years. The only variables included in the model during the final step were sex and BMI at age 5. The sensitivity, specificity, and predicted precision of the decision to treat individuals was then calculated for cut-off points representing integer values of BMI between 16 and 20 at age 5. The best performing BMI cut-off points identified using the derivation cohort data were considered for inclusion in the decision protocol (table 4).

**Table 4:** Diagnostic accuracy at the age 10 of obesity prediction at age 5 using cut-off points at BMI values 16–20.

BMI cut-off value at age 5	Fraction chosen for treatment (%)	Sensitivity (%)	Specificity (%)	Precision of treatment choice (%)	OR	95% CI
*Girls*						
16	39.9	95	62	9	29.8	7.1–124.6
17	18.6	89	84	19	45.7	16.0–130.6
18	8.4	71	94	33	39.6	18.7–84.1
19	4.4	60	98	53	70.8	32.2–155.5
20	2.1	37	99	67	78.0	28.9–210.7
*Boys*						
16	43.1	93	60	11	18.9	6.8–52.7
17	18.5	80	85	23	22.5	11.4–44.6
18	7.3	53	95	39	22.5	12.3–41.3
19	3.4	42	99	66	57.7	26.4–126.1
20	2.0	29	99	76	79.6	27.7–228.3

OR = odds ratio

95% CI = 95% confidence interval

The variables in final inductive model extracted from the derivation cohort using stepwise logistic regression were BMI at age 5 and sex. Variables not included in the model were BMI at the ages 1½, 2½, and 4 years, age at adiposity rebound, BMI change, BMI at adiposity rebound, and early adiposity rebound.

### Protocol accuracy

**Figure 2:**
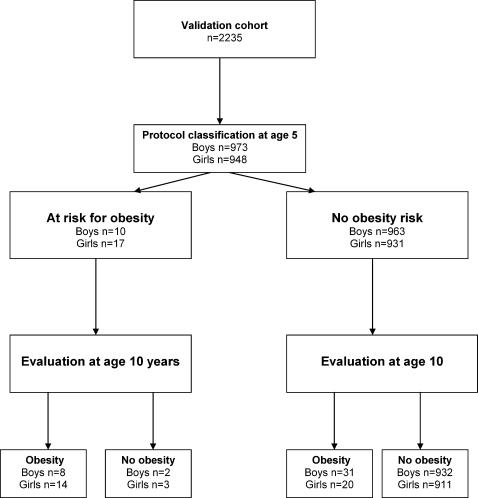
Protocol assessment according to the STARD procedure.

The decision rule chosen for boys was to treat those identified with obesity at checkups at ages 4 and 5 years. For girls, the cut-off point at BMI 20 from the inductive model was chosen for the decision protocol. The decision protocol selected 1.4% of the preschool children as being at risk for persisting obesity (figure 2). The decision accuracy of the protocol at age 10 is shown in table 5. While the specificity (100%) and the precision (80–82%) of the protocol were similar for boys and girls, the sensitivity was almost twice as high for girls (41%) than for boys (21%). The relative risk for obesity at age 10 estimated by the odds ratio for the selected children compared to non-selected peers was 212.6 (95% CI 56.6 to 798.4) for girls and 120.3 (95% CI 24.5 to 589.9) for boys.

**Table 5:** Diagnostic accuracy of decision protocol to be used at the age 5 checkup for selection of children at risk for persisting obesity.

	Fraction chosen for treatment (%)	Sensitivity (%)	Specificity(%)	Precision of treatment choice (%)	OR	95% CI
**Decision protocol**						
*Boys*						
Obesity at 4- and 5-year checkups	1.0	21	100	80	120.3	24.5–589.9
*Girls*						
BMI above 20 at 5-year checkup	1.8	41	100	82	212.6	56.6–798.4
**Rules excluded from protocol**						
*Girls*						
Obesity at 4- and 5-year checkups	1.8	33	99	69	85.1	27.2–265.7
Obesity at either 4 or 5-year checkup	3.3	55	99	60	84.4	34.6–205.8
Obesity at 5-year checkup	3.3	52	99	59	74.7	30.7–181.8
BMI above 16 at 5-year checkup	38.9	100	64	10	–	–
BMI above 17 at 5-year checkup	19.1	85	83	16	29.1	11.1–76.3
BMI above 18 at 5-year checkup	8.9	71	93	29	34.2	15.6–74.7
BMI above 19 at 5-year checkup	4.1	53	98	46	47.8	21.5–106.5
*Boys*						
Obesity at either 4 or 5-year checkup	2.7	33	99	48	32.9	14.0–76.8
Obesity at 5-year checkup	2.1	31	99	57	45.7	17.7–117.5
BMI above 16 at 5-year checkup	42.0	93	60	9	21.2	6.5–68.9
BMI above 17 at 5-year checkup	18.7	82	84	19	24.5	11.2–53.7
BMI above 18 at 5-year checkup	7.2	58	93	34	26.2	13.6–50.6
BMI above 19 at 5-year checkup	2.7	38	99	61	55.1	23.6–128.4
BMI above 20 at 5-year checkup	1.6	22	99	59	40.9	14.7–113.8

OR = odds ratio

95% CI = 95% confidence interval

## Discussion

We found that obesity according to IOTF criteria at both age 4 and age 5 checkups in boys, and a BMI 20 or above at the age 5 checkup for girls were strongly associated with obesity at age 10. These findings were used to construct a clinical protocol for selection of preschool children at high risk for obesity persisting into preadolescence and adolescence. The protocol was assessed for use at the final well child control at age 5 and found to display high precision and specificity, while the sensitivity was low especially for boys. Even though the protocol can help to identify with high precision 33% of the children being obese in preadolescence among only 1.4% of the children starting school at age 6, it must be taken into account that 67% of obese preadolescents are missed. The identification of this group at preschool age warrants further research. We examined different ways of increasing protocol performance by assessing all factors that at present were routinely taken into regard at the checkups at Swedish child health centres, including adiposity rebound and rapid BMI increase, but these parameters did not improve efficacy. Comprising more parameters that have been reported to be associated with childhood obesity in the protocol development could have improved the decision accuracy, e.g. parent weight, genetic factors, and physical activity level.[24] However, in Sweden (population 9 million), each annual birth cohort comprises about 100.000 children. Based on that each child is seen at the child health centre six times, any additional routine laboratory test would cause substantial costs, and collection of clinical data in standardized form on specific factors, such as parents' BMI, would require considerable efforts from the practitioners. There are still variables that can be used to improve the sensitivity of the protocol, also when taking cost-effectiveness issues into regard. This set of variables comprises, e.g. birth weight data [25], the length of the breastfeeding period [26], and eating patters [27]. The gradual extension of the protocol with these variables can lead to that fewer obese preadolescents are missed, while retaining the high precision and cost-effectiveness.

### Protocol transfer to other child health settings

A slightly higher prevalence of adiposity was observed in our cohort at age 10 than was recently reported from a similar setting in Western Sweden,[3] while a study of Norwegian fourth graders[28] showed rates similar to ours. Recent studies indicate that the major development of early adiposity is established before puberty.[4], [5], [29] We therefore used obesity at age 10 as the reference standard for persisting adiposity. The current references for identification of children at risk for persistent obesity are the BMI chart cut-off points. We found that the IOTF cut-off points displayed a lower precision at the age 5 checkup, 57% for boys and 59% for girls, than the protocol (80%–82%). The main reason the protocol performs better than the IOTF cut-off is because it has been optimised with regard to the age 5 checkup. For boys the BMIs at two ages (4 and 5 years) was combined, which reduced measurement error and hence misclassification. For girls, a more extreme cut-off than the IOTF was used at age 5, simply because it performed better (Tables 3 and 5). The fact that the protocol had higher precision for girls can be explained partly by the fact that boys more often engage in sports and exercise at ages 6–10,[30] and partly due to hormonal differences during the age period.

### Trajectory versus point measurements

Variables reflecting rapid BMI change and early adiposity rebound have in previous studies[31] been associated with risk for persistent obesity, but in this study they did not qualify for inclusion in the final inductive model. This discrepancy can be explained by the fact that studies restricted to examination of a few factors can seldom account for the relative impact of these factors on the development of persisting obesity.[32] In this study, we analysed several trajectory and point measurement variables derived from longitudinal recordings of weight and height during the preschool period that were routinely available at the age 5 checkup. When compared to point measurements, we found that the only trajectory component included in the protocol was a history of obesity in boys according IOTF cut-off points at ages 4 and 5. It is possible that a more detailed definition of the trajectory variables may have increased their influence on the prediction of obesity, but such constructions would also have made the variables less practical for use at child health centres.

### Protocol application in child health practice

A shortcoming of the protocol is that the selected children are already obese. An optimal protocol would be one that selects for prevention the still non-obese preschool children that are at high risk for later obesity. Nevertheless, present results suggest that the development of such a protocol would necessitate collection of a larger data set, probably including variables that cover foetal exposures [33] and data about the inherited bio-behaviour system [34]. It must also be acknowledged that motivating families with normal-weight children to comply with obesity prevention program poses additional challenges.

Overall, we found that the protocol performed sufficiently well to qualify for effectiveness studies. It has a clearly formulated purpose, is concise, and should be easy to use at child health centres. School nurses with no knowledge of the protocol assessed the outcome parameter, i.e. obesity at age 10. While the decision rule and inductive model components were derived in a standardised fashion from a randomly selected non-biased population of children, the classification performance was demonstrated on a new population. Previous studies indicate that clinical impression is the current ‘gold standard’ for deciding whether or not children are at risk for persistent obesity.[13] We believe that the present protocol can contribute to the refinement of this standard towards an evidence-based management procedure. The definitions of overweight and obesity that have previously been used for selecting children for prevention programmes have seldom been associated with estimates of risk for persistent obesity. Our experience is that low motivation can be a major problem when approaching parents with children found at risk for persistent obesity with treatment recommendations, and this experience has also been confirmed in other settings.[35] The performance record of the protocol can be used during the age 5 checkup when informing the selected children's parents, i.e. that their child is at high risk for persistent obesity, that there are promising family-based interventions available, and that these interventions will require involvement from the entire family during a long period of time. The performance record of the protocol may both increase motivation to participate in interventions and motivate more parents to comply with therapy over time.
